# The Symptoms of Locomotor Ataxia

**Published:** 1903-04-04

**Authors:** 


### THE SYMPTOMS OF LOCOMOTOR ATAXIA.

In the *New York Medical News* of March 7th and 14th Dr. Collins presents a detailed consideration of the various symptoms met with in locomotor ataxia. The most general of these symptoms is pain in some form or another. It is present in 90 per cent. of all cases of tabes dorsalis, and is the initial symptom in 40 per cent. The pain has various characters, and is not of a stereotyped kind. In 67 per cent. of cases it affects the lower extremities. Visceral crises occurred in 14 per cent. of Dr. Collins' 140 cases. The organs to which the attacks were referred were as follows: stomach, 11 cases; rectum, four cases; larynx, two cases; intestines, one case; larynx and pharynx, one case; stomach and intestines, one case. In six of these 20 cases the crises were the initial symptoms; in such cases the illness is rarely correctly diagnosed until it has lasted a considerable length of time. Paræsthesia is one of the commonest symptoms: its occurrence was noted in 73 per cent. of Dr. Collins' cases. The symptom is described in a variety of ways by the patients. The commonest designations are numbness, tingling, pins and needles, and so forth. The lower extremities are the parts most often affected. Paræsthesia is an important symptom in nearly every case of tabes dorsalis. Disturbance of tactile sensibility is an exceedingly frequent symptom, and as it is usually present at a very early stage of the disease, it is often of considerable value in assisting the diagnosis. The trunk and lower extremities are the usual sites, but the sensory disturbances may occur in any part of the cutaneous area; in two cases the face gums, teeth, and tongue were anæsthetic. Diminution of tactile sensation over certain areas is liable to be overlooked unless careful systematic examination is carried out with a camel's-hair brush or some similar soft substance. The commonest situation for impairment of tactile sensation to be found early in the disease is on the trunk, in the area corresponding to the fourth and sixth dorsal segments. The distinguishing feature of the tactile disturbance is that it corresponds in the area affected to the sensory distribution of segments of the spinal cord. Analgesia was noted in 58 per cent. of Dr. Collins' cases. In his experience it is of frequent occurrence in the areas of skin supplied by the ulnar and peroneal nerves. In 140 cases ulnar analgesia was noted 34 times and peroneal analgesia 43 times; in many of these cases it was present in both areas. Careful examination into the tonus of the muscles was made in 57 cases, and in 41 of these (72 per cent.) some degree of hypotonus was found. As a rule it was found in a much more advanced degree in patients in which the disease had existed for a long time. In some cases it was comparatively slight, i.e., with the leg extended the thigh could only be brought to a little less than a right angle with the body, the patient lying flat on the back. In other instances the leg could be approximated to the shoulder. Although the motor symptoms are those from which the disease takes its name they are rarely conspicuous in the early stages of the complaint. In fact the disease may exist for many years without causing noticeable impairment of the motor apparatus. Ataxia develops sooner or later in every instance if the patient lives long enough. Ataxia of gait was present in 73 per cent. of the cases which Dr. Collins examined. In the majority of cases the symptom was first noticed by the patient when crossing a crowded thoroughfare, walking in a narrow space, or going downstairs. The inco-ordination may appear quite suddenly in a man who has suffered for years from other symptoms of tabes dorsalis such as pains, visceral crises, etc. In the very early stages of the affection the knee jerks are present, later they become sluggish and disappear. They may during the intermediate stage be present on one side but absent on the other. Babinski's phenomenon was not elicited in one out of 41

instances where the sign was specially looked for. Ocular palsies are fairly frequent, and when they occur it is as a rule at a comparatively early stage of the disease, and the diplopia complained of is usually intermittent in its manifestation. The third nerve is the one most commonly affected. With regard to the occurrence of optic atrophy in tabes, Dr. Collins has not noticed that the course of the affection is less severe in those cases where optic atrophy is present than in those where it is absent. In this his experience differs from many writers on the subject. The pupillary phenomena of tabes are inequality in size of the two pupils and the Argyll-Robertson pupil, though the reaction of the pupil during accommodation may be lost as well as the light reflex. Dr. Collins has worked out the comparative frequency of the most important symptoms as they were observed in his own cases; his results expressed as percentages are as follows: lancinating pain, 90; Romberg's sign, 85; loss of ankle jerk, 88; loss of knee jerk, 84; Argyll-Robertson pupil, 77; ataxia of gait, 73; hypotonia, 72; paræsthesia, 73; anæsthesia, 67; analgesia, 58; bladder symptoms, 55; impotency, 50; crises, 20; ocular paralysis, 10; arthropathies, 5; trophic symptoms, 8; optic atrophy, 14.

